# Open-label, multicenter, single-arm phase II DeCOG-study of ipilimumab in pretreated patients with different subtypes of metastatic melanoma

**DOI:** 10.1186/s12967-015-0716-5

**Published:** 2015-11-06

**Authors:** Lisa Zimmer, Thomas K. Eigentler, Felix Kiecker, Jan Simon, Jochen Utikal, Peter Mohr, Carola Berking, Eckhart Kämpgen, Edgar Dippel, Rudolf Stadler, Axel Hauschild, Michael Fluck, Patrick Terheyden, Rainer Rompel, Carmen Loquai, Zeinab Assi, Claus Garbe, Dirk Schadendorf

**Affiliations:** Department of Dermatology, University Hospital, University Duisburg-Essen, Essen, Germany; Department of Dermatology, Center for Dermatooncology, University Medical Center Tübingen, Tübingen, Germany; Department of Dermatology and Allergy, Skin Cancer Center, Charité-Universitätsmedizin Berlin, Berlin, Germany; Department of Dermatology, Venereology and Allergology, University Hospital Leipzig, Leipzig Germany,; Skin Cancer Unit, German Cancer Research Center (DKFZ), Heidelberg, Germany; Department of Dermatology, Venereology and Allergology, University Medical Center Mannheim, Ruprecht-Karl University of Heidelberg, Mannheim, Germany; Department of Dermatology, Elbekliniken Stade Buxtehude, Buxtehude, Germany; Department of Dermatology and Allergy, Ludwig-Maximilian University, Munich, Germany; Department of Dermatology, Dermatologikum Berlin, Berlin, Germany; Department of Dermatology, Klinikum Ludwigshafen, Skin Cancer Center Rheinpfalz, Ludwigshafen, Germany; Department of Dermatology, Medical Centre Minden, Minden, Germany; University Department of Dermatology, Kiel, Germany; Department of Dermatology Hornheide, Münster, Germany; Department of Dermatology, University of Lübeck, Lübeck, Germany; Department of Dermatology, Clinical Centre Kassel, Kassel, Germany; Department of Dermatology, University Medical Center, Johannes Gutenberg-University, Mainz, Germany

**Keywords:** Phase II, Melanoma, Cutaneous melanoma, Mucosal melanoma, Occult melanoma, Ipilimumab, Anti-CTLA-4

## Abstract

**Background:**

Ipilimumab is an approved immunotherapy that has shown an overall survival benefit in patients with cutaneous metastatic melanoma in two phase III trials. As results of registrational trials might not answer all questions regarding safety and efficacy of ipilimumab in patients with advanced melanoma seen in daily clinical practice, the Dermatologic Cooperative Oncology Group conducted a phase II study to assess the efficacy and safety of ipilimumab in patients with different subtypes of metastatic melanoma.

**Patients and methods:**

We undertook a multicenter phase II study in melanoma patients irrespective of location of the primary melanoma. Here we present data on patients with pretreated metastatic cutaneous, mucosal and occult melanoma who received up to four cycles of ipilimumab administered at a dose of 3 mg/kg in 3 week intervals. Tumor assessments were conducted at baseline, weeks 12, 24, 36 and 48 according to RECIST 1.1 criteria. Adverse events (AEs), including immune-related AEs were graded according to National Cancer Institute Common Toxicity Criteria (CTC) v.4.0. Primary endpoint was the OS rate at 12 months.

**Results:**

103 pretreated patients received at least one dose of ipilimumab, including 83 cutaneous, seven mucosal and 13 occult melanomas. 1-year OS rates for cutaneous, mucosal and occult melanoma were 38 %, 14 % and 27 %, respectively. Median OS was 6.8 months (95 % CI 5.3–9.9) for cutaneous, 9.6 months (95 % CI 1.6–11.1) for mucosal, and 9.9 months (lower 95 % CI 2.3, upper 95 % CI non-existent) for occult melanoma. Overall response rates for cutaneous, mucosal and occult melanoma were 16 %, 17 % and 11 %, respectively. Eleven patients had partial response (16 %) and ten patients experienced stable disease (14 %), none achieved a complete response. Treatment-related AEs were observed in 71 patients (69 %), including 20 grade 3–4 events (19 %). No new and unexpected safety findings were noted.

**Conclusions:**

Ipilimumab is a treatment option for pretreated patients with advanced cutaneous melanoma seen in daily routine. Toxicity was manageable when treated as per protocol-specific guidelines.

Trial registration: Clinical Trials.gov NCT01355120

**Electronic supplementary material:**

The online version of this article (doi:10.1186/s12967-015-0716-5) contains supplementary material, which is available to authorized users.

## Background

From a clinical like from a scientific perspective, the recent advances in cancer immunotherapy have been acknowledged as a major breakthrough [1]. Especially for melanoma patients, immune checkpoint inhibitors begin to witness an enormous therapeutic potential, resulting very recently in the approval of the first-in-class anti-programmed-death-receptor-1 (PD-1) inhibitors nivolumab and pembrolizumab for the treatment of unresectable or metastatic melanoma [2–5].

The cytotoxic T-lymphocyte-associated antigen-4 (CTLA-4) inhibitor ipilimumab has been authorized for use in advanced, metastatic melanoma in the United States and in the European Union—as in many other countries worldwide since—on basis of two pivotal phase III studies [6, 7]. CTLA-4, a native regulator of T cell activation, downregulates T-cell function through a variety of mechanisms, and finally induces T-cell cycle arrest [8]. Because many of the immune checkpoints are regulated by ligand-receptor interactions, CTLA-4 can be easily blocked by monoclonal antibodies or recombinant ligand-like proteins that block CTLA-4 as a negative regulator of immunity, hence enhancing natural antitumor immunity [9].

Serving as the first mechanistically defined immune checkpoint inhibitor, ipilimumab has been intensely investigated in clinical registrational trial settings [6, 7] in patients with cutaneous melanoma, the most frequent melanoma subgroup with morphological and molecular distinctions from other clinical disease subgroups [10]. However, results of registrational trials might not answer all questions regarding safety and efficacy of ipilimumab in advanced melanoma patient cohorts seen in daily routine.

Here we report the results of the open-label, multicenter, single-arm phase II DeCOG trial to further evaluate the efficacy and safety of 3 mg/kg ipilimumab in pretreated patients with cutaneous, mucosal and occult metastatic melanoma seen in daily routine in interdisciplinary skin cancer units in Germany. Data for patients with ocular melanoma are reported elsewhere [11].

## Patients and methods

### Patients

Eligibility criteria included documented unresectable stage III or stage IV metastatic cutaneous, occult, mucosal and ocular melanoma according to American Joint Committee on Cancer cutaneous melanoma staging criteria [12]. Patients who had received at least one prior systemic therapy were eligible. Previous systemic treatment had to be completed ≥28 days before receiving ipilimumab. Additional requirements included age ≥18 years, Eastern Cooperative Oncology Group (ECOG) performance status ≤2, life expectancy of ≥6 months (estimation of life expectancy was at the discretion of the participating investigators), measurable disease according to Response Evaluation Criteria In Solid Tumors (RECIST) 1.1 [13], adequate bone marrow, renal and hepatic function. Patients with a history of active autoimmune disease and chronic use of systemic corticosteroids were excluded. Patients with asymptomatic, radiographically stable previously treated or untreated brain metastases were eligible.

### Study design

This multicenter, open-label, phase II study (DeCOG-MM-PAL11-Trial; CA184-137) was conducted in two parts. Part 1 of the study was open for recruitment from May 2011 to August 2011; in an Additional file 1: Figure S1 the patient flow is described. This part allowed recruitment of pretreated melanoma patients irrespective of location of the primary melanoma. Part 2, which was only eligible for patients with pretreated or treatment-naïve metastatic ocular melanoma to allow for a valid analysis of this subgroup, was closed on September 30, 2012. Data from part 1 and 2 for patients with ocular melanoma are reported elsewhere [11]. Twenty-five Dermatologic Cooperative Oncology Group (DeCOG) skin cancer units in Germany participated. The study was approved by institutional ethics committee University Duisburg-Essen (approval number 10–4531) and the German competent authority Paul-Ehrlich-Institute (Langen, Germany, approval number 1233), and conducted in accordance with the Declaration of Helsinki/Good Clinical Practice. All patients gave written informed consent. The protocol for this trial is available as Additional file 2. Ipilimumab was administered intravenously over 90 min at a dose of 3 mg/kg every 3 weeks for a total of four infusions. Patients with progressive disease (PD) at ≥3 months from week 12 assessment following stable disease (SD), an initial partial (PR) or complete response (CR) were eligible for re-induction with ipilimumab following at the same dosage. Dose reduction was not allowed, but skipping of one dose of ipilimumab was recommended when adverse events (AE) occurred. Rapid disease progression, intolerable toxicity or patient withdrawal led to treatment discontinuation. The primary endpoint was the overall survival (OS) rate at 12 months.

### Assessments

Regular assessments, including a physical examination and standardized blood testing, were carried out at baseline and every 3 weeks during induction and re-induction phases. Tumor assessments were conducted at baseline, weeks 12, 24, 36 and 48 using the RECIST version 1.1 [13]. Adverse events (AEs) were graded according to the National Cancer Institute Common Toxicity Criteria (CTC version 4.0). All AEs were recorded from the time of the first ipilimumab administration until 70 days after treatment discontinuation. AEs were defined as an immune-related AE (irAE) if they were associated with drug exposure, consistent with an immune phenomenon and if other causes were ruled out. IrAE management was based on protocol-specific treatment algorithms. All AEs that were definitely, probably or possibly related to study drug were defined as related AEs.

### Statistical methods

This report includes results based on the data cutoff of December 6, 2013. Patient and disease characteristics were analyzed using descriptive statistics. Categorical values were expressed as counts and percentage whereas continuous values were expressed as median and range values. OS was defined as the time from the first administration of ipilimumab to death from any cause. Patients last known to be alive were censored at the date of last contact. Progression-free survival (PFS) was defined as the time from the first dose of ipilimumab to the first date of documented progression as per RECIST, or date of death, whichever came first. Patients last known to be alive and progression-free were censored at the date of last contact. PFS rate at 6 months was defined as the proportion of patients being alive and without progress 6 months after the first ipilimumab administration. Patients with unknown survival status or unknown status of progression at 6 months were censored. The 1- and 2-year survival rates were defined as the proportion of patients being alive 12 or 24 months after their first ipilimumab administration. Patients with unknown survival status at 12 or 24 months were censored. OS, PFS, PFS rate at 6 months, 1- and 2-year survival rates were estimated by the Kaplan–Meier method. For medians of OS and PFS, 95 % confidence intervals (CIs) were calculated using the Brookmeyer and Crowley method. The log-rank test was used to compare the 1-year and 2-year OS rates in patients with cutaneous melanoma between several subgroups, i.e. the BRAF mutational status, the presence of brain metastases, the lactate dehydrogenase (LDH) level prior to receiving ipilimumab [<2-fold upper level norm (ULN) vs. ≥2× ULN], the number of ipilimumab doses (<4 vs. 4), and the absolute lymphocyte count (ALC) (<1000/µl vs. ≥1000/µl) before the first (week 1), the second (week 4) and the third dose (week 7) of ipilimumab. Due to small sample sizes comparisons of 1-year and 2-year OS rates in patients with mucosal and occult melanoma were not done. Two sided p values were evaluated and a p value of <0.05 was considered statistically significant. All variables with significant differences between their stratifications regarding the overall survival were included in a multivariate Cox proportional hazards model. To determine potential predictors, all independent covariates (LDH, number of ipilimumab doses, ALC week 4, brain metastases), were entered into a backward Cox regression model for the overall survival. The stay level was p = 0.05. All covariates being still significant were considered as potential predictors. For the hazard ratio, 95 % CIs were calculated using the Wald method. The overall response rate (ORR) was defined as the proportion of patients with PR and CR whereas the disease control rate (DCR) was defined as the proportion of patients with CR, PR and SD. Lost to follow-up was documented if the patient did not respond to phone calls (3 times) and to a written invitation. Analyses were carried out using SAS software, version 9.3 (Cary, NC, USA).

## Results

### Patients

Between May to August 2011, 103 patients were enrolled and received at least one dose of ipilimumab, including 83 patients with cutaneous melanoma, 13 with occult melanoma and seven with mucosal melanoma (Table 1). Baseline patient characteristics are reported in Table 1. All 103 patients had received previous systemic anti-cancer treatment, including chemotherapy, immunotherapy, and targeted agents (Table 1). The most common chemotherapies were dacarbazine and carboplatin/paclitaxel given in 73 (71 %) and 30 (29 %) of all patients. None had previously received ipilimumab but 18 patients had undergone previous immunotherapy treatment with interferon α or vaccination. 31 patients presented with brain metastases at study entry, with similar proportions observed across the different melanoma subtypes (Table 1). Sixty-four patients (62 %) completed the induction phase, including 52 patients with cutaneous, four with mucosal and eight with occult melanoma. Three patients with cutaneous melanoma experienced PR at week 12 and were re-induced after 91, 232 and 217 days, respectively (Table 2). The median number of doses received in the induction phase was four (range 1–4). Among the 39 patients (38 %) who did not complete the induction phase, 11 (11 %) died, 16 (16 %) developed PD, eight (8 %) had intolerable AEs and four (4 %) withdrew their informed consent.

**Table 1:** Baseline patient characteristics (n = 103 patients totally)

Patient characteristics	Cutaneous melanoma		Mucosal melanoma		Melanoma of unknown primary	
	N	%	N	%	N	%
*No. patients, %*	83	100	7	100	13	100
*Age, years*						
Median (range)	63	(29–85)	63	(33–37)	62	(40–77)
*Sex*						
Male	53	64	2	29	11	29
Female	30	36	5	71	2	71
*ECOG baseline*						
0	51	61	2	29	12	92
1	23	28	5	71	1	8
2	9	11	–		–	
*BRAF mutation*						
Not mutated	29	35	3	43	5	39
Mutated	17	21	–		6	46
Not known	37	45	4	57	2	15
*Disease stage (all: Stage IV)*						
M1a	6	7	–		3	23
M1b	15	18	2	29	1	8
M1c	62	75	5	71	9	69
*LDH*						
<2 ULN	67	81	5	71	11	85
≥2 ULN	16	19	2	29	2	15
*Brain metastases*						
No	57	69	5	71	10	77
Yes	26	31	2	29	3	23
*Prior systemic therapy in stage IV (except radiotherapy)*						
No	–		–		–	
Yes	83	100	7	100	13	100
*Number of prior systemic therapies*						
1	42	51	6	86	8	62
2	27	33	–		3	23
≥3	13	16	1	14	2	15
Not applicable	1	1	–		–	
*Immunotherapy*						
No	67	81	7	100	11	85
Yes	16	19	–		2	15
*If yes, type of previous immunotherapy*						
Interferon alpha	11	13	–		2	15
Vaccination	5	6	–		–	
Kinase inhibitors						
No	71	86	7	100	9	69
Yes	12	14	–		4	31
*If yes, type of previous kinase inhibitor*						
BRAF inhibitor	7	8	–		2	15.5
MEK inhibitor	4	5			2	15.5
*Chemotherapy*						
0	9	11	–		2	15
1	47	57	6	86	8	62
2	20	24	–		2	15
≥3	7	8	1	14	1	8

*ECOG* Eastern Cooperative Oncology Group, *LDH* lactate dehydrogenase

**Table 2:** Outcomes of patients with ipilimumab re-induction therapy

Age, years	Best response at week 12 (RECIST)	Duration between 1st restaging (week 12) and re-induction therapy (days)	Response at 1st restaging after re-induction (RECIST)	Best overall response after re-induction (RECIST)	Time from 1st dose to death/follow-up (months)	Alive
74	PR	91	SD	SD	17.1	Yes
56	PR	232	PR	CR	25.5	Yes
73	PR	217	PD	SD	24.8	Yes

*RECIST* response evaluation criteria in solid tumors, *PR* partial response, *SD* stable disease, *PD* progressive disease

### Efficacy

**Fig. 1:**
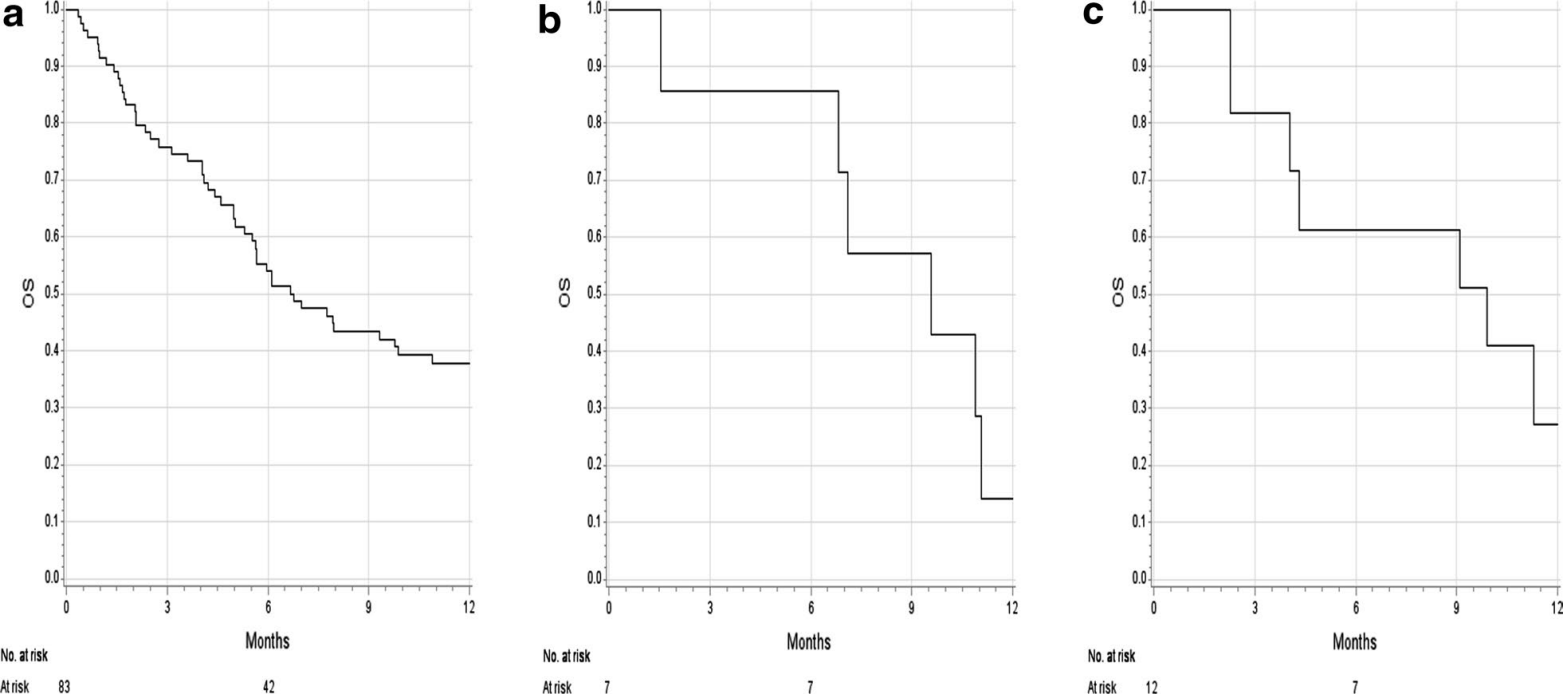
Kaplan–Meier curves for 1-year overall survival (OS) rates of different melanoma subtypes. Pretreated patients with **a** metastatic cutaneous melanoma (1-year OS rate: 38 %), **b** mucosal melanoma (1-year OS rate: 14 %), and **c** occult melanoma (1-year OS rate: 27 %). All patients received ipilimumab 3 mg/kg

The 1-year rate for OS was 38 % (95 % CI 27–49) for cutaneous melanoma, 14 % (95 % CI 1–47) for mucosal melanoma, and 27 % (95 % CI 5–57) for occult melanoma. 2-year OS rates for cutaneous and occult melanoma were 22 % (95 % CI 13–33) and 27 % (95 % CI 5–57), respectively. All of the patients with mucosal melanoma died before month 24 after the first ipilimumab administration. Six-month rate for PFS were 16 % (95 % CI 9–25) for cutaneous melanoma, 14 % (95 % CI 1–47) for mucosal melanoma, and 17 % (95 % CI 3–41) for occult melanoma. Median OS from the first dose of ipilimumab for cutaneous, mucosal and occult melanoma were 6.8 (95 % CI 5.3–9.9; Fig. 1a), 9.6 (95 % CI 1.6–11.1; Fig. 1b) and 9.9 (lower 95 % CI 2.3, upper 95 % CI non-existent; Fig. 1c) months, respectively. Seventy of 103 patients were evaluable for efficacy assessment (Table 3). Among the 33 patients (32 %) who were not assessable, 22 died before the assessment of change in tumor burden (including 13 with brain metastases), three developed PD (including two with brain metastases), three had intolerable AEs, one had no measurable disease at baseline, three withdrew their informed consent (including one with brain metastases) and one was lost to follow-up. The DCR was 29 % for cutaneous, 50 % for mucosal and 22 % for occult melanoma (Table 3). Overall response rates for cutaneous, mucosal and occult melanoma were 16 %, 17 % and 11 %, respectively (Table 3). Among the 70 patients evaluable for response, the overall response rate for 15 patients with brain metastases was 13 %: a response rate similar to the one found for the remaining 55 patients without brain metastases (16 %). Of the 15 patients with brain metastases seven patients had intracranial SD, seven intracranial PD and one patient experienced intracranial CR. In total, ten patients showed similar response pattern in intracranial and extracranial metastases and five patients had different response pattern, e.g. in one patient an intracranial response (CR) was observed, unfortunately associated with extracranial PD (Additional file 3: Table S3).

**Table 3:** Overall response rates (ORR) and disease control rates (DCR) (n = 70 patients totally)

Patients with measurable disease (and at least one tumor assessment)	Cutaneous melanoma		Mucosal melanoma		Melanoma of unknown primary	
	N	%	N	%	N	%
*No. patients (%)*	55	100	6	100	9	100
*Response pattern (acc. to RECIST)*						
Complete response	–		–		–	
Partial response	9	16	1	17	1	11
Stable disease	7	13	2	33	1	11
Progressive disease	39	71	3	50	7	78
*Best ORR (according to RECIST)*						
ORR (=CR + PR)	9	16	1	17	1	11
ORR at week 12	7	13	1	17	–	
ORR at week 24	6	11	1	17	–	
*Best DCR (according to RECIST)*						
DCR (=CR + PR + SD)	16	29	3	50	2	22
DCR at week 12	15	27	3	50	2	22
DCR at week 24	10	18	1	17	1	11

*CR* complete response, *PR* partial response, *RECIST* response evaluation criteria in solid tumors, *SD* stable disease

**Fig. 2:**
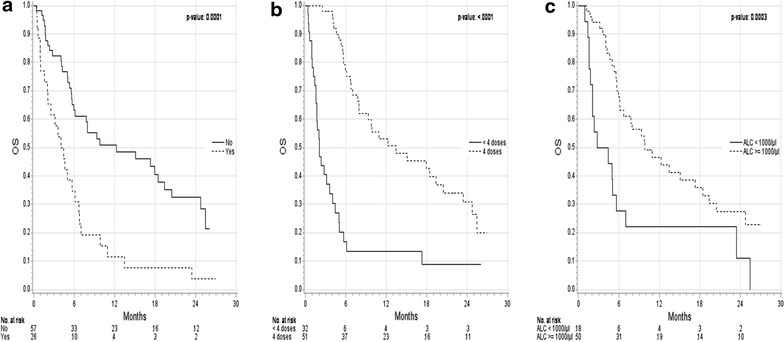
Kaplan–Meier curves for overall survival (OS) of subgroups (pretreated patients with metastatic cutaneous melanoma). Subgroups were stratified as follows: by **a** the absence of brain metastases before the first dose of ipilimumab; Absence of brain metastases: median OS 12.3 months (95 % CI 6.0–19.4); brain metastases present: median OS 4.2 months (95 % CI 2.0–6.1); **b** the number of ipilimumab doses (4 versus <4); 4 doses: median OS 13.5 months (95 % CI 7.9–20.4); <4 doses: median OS 2.1 months (95 % CI 1.6–4.1); and **c** the absolute lymphocyte count (ALC) (≥1000/µl versus <1000/µl) before the second dose (i.e. week 4) of ipilimumab; ALC ≥1000/µl: median OS 9.9 months (95 % CI 6.1–18.5); ALC <1000/µl: median OS 3.6 months (95 % CI 1.8–5.6)

The 1-year OS rate was higher in patients with cutaneous melanoma who had no brain metastases (51 % vs. 12 %, p < 0.0001, Fig. 2a), in patients with a LDH level <2× ULN (42 % vs. 19 %, p = 0.0007), in patients who received four ipilimumab doses (53 % vs. 14 %, p < 0.0001; Fig. 2b), and in patients with an ALC ≥1000/µl before the second dose of ipilimumab (week 4) (47 % vs. 22 %, p = 0.002; Fig. 2c). The apparent better OS observed in patients who received all four ipilimumab doses, could be solely based on a time dependent bias, as receiving four doses of ipilimumab required surviving >10 weeks after therapy initiation. This only applied to 39 % of the patients who received <4, but not surprisingly all with four doses of ipilimumab. BRAF mutational status, the ALC before the first and the third dose of ipilimumab in patients with cutaneous melanoma were not associated with OS. In a multivariate analysis, the factors independently associated with better OS were the administration of four ipilimumab doses (e.g. patients with less than 4 doses were at higher risk of death; hazard ratio 4.3, 95 % CI 2.3–8.0), an ALC ≥1000/µl before the second dose of ipilimumab (week 4) (e.g. patients with ALC <1000/µl were at higher risk of death; hazard ratio 2.0; 95 % CI 1.1–3.8), and the absence of brain metastases (e.g. patients with brain metastases were at higher risk of death; hazard ratio 1.9, 95 % CI 1.0–3.5).

### Safety

Ninety-eight of 103 patients (95 %) experienced one or more AEs (Table 4). Treatment-related AEs were reported in 71 patients (69 %); 20 patients (19 %) had treatment-related grade 3 or 4 AEs. The majority of treatment-related AEs were irAEs, occurring in 52 patients (51 %). Most common irAEs were gastrointestinal disorders—diarrhea and colitis, skin-related toxic effects—pruritus and rash, and hepatic disorders—increased alanine aminotransferases (ALT) and aspartate aminotransferases (Table 4). The most frequent grade 3 or 4 irAEs were diarrhea and colitis, noted in ten (10 %) and 5 patients (5 %), respectively. There was one patient with a gastrointestinal perforation due to grade 3 colitis and diarrhea. After surgery and treatment with 2 mg/kg methylprednisolone intravenous, diarrhea and colitis improved and corticosteroid therapy was tapered slowly over 6 weeks. Immune-related AEs were generally reversible when managed as per protocol-specific treatment guidelines. Most of the irAEs resolved with corticosteroid therapy. None of the patients required additional immunosuppression with infliximab or mycophenylate mofetil. Treatment related non-irAEs included anemia, fatigue, bone pain, fever, nausea and vomiting. There was no treatment-related death.

**Table 4:** Reported adverse events in overall study population (n = 103 patients totally)

Adverse events (AE)^a^	Cutaneous melanoma		Mucosal melanoma		MUP		Total	
No. patients (%)	83 (100)		7 (100)		13 (100)		103 (100)	
	All grades	Grade 3/4	All grades	Grade 3/4	All grades	Grade 3/4	All grades	Grade 3/4
*Patients with at least one AE*	79 (95)	36 (43)	6 (86)	4 (57)	13 (100)	7 (54)	98 (95)	47 (46)
*Patients with treatment-related AE*	57 (69)	14 (17)	3 (43)	2 (29)	11 (85)	4 (31)	71 (69)	20 (19)
*Patients with any irAE*	40 (48)	12 (15)	2 (29)	1 (14)	10 (77)	4 (31)	52 (51)	17 (17)
*irDermatitis*	21 (25)	–	1 (14)	–	3 (23)	–	25 (24)	–
Pruritus	8 (10)	–	1 (14)	–	2 (15)	–	11 (11)	–
Rash	8 (10)	–	–	–	1 (8)	–	9 (9)	–
Erythema multiforme	4 (5)	–	–	–	–	–	4 (4)	–
Hand-foot-syndrome	1 (1)	–	–	–	–	–	1 (1)	–
*irGastrointestinal disorders*	39 (47)	15 (18)	2 (28)	1 (14)	8 (62)	4 (31)	49 (48)	20 (20)
Colitis	6 (7)	4 (5)	–	–	1 (8)	1 (8)	7 (7)	5 (5)
Diarrhea	25 (30)	8 (10)	1 (14)	1 (14)	4 (31)	1 (8)	30 (29)	10 (10)
GI-perforation	1 (1)	1 (1)	–	–	–	–	1 (1)	1 (1)
Other^b^	7 (9)	2 (2)	1 (14)	–	3 (23)	2 (15)	11 (11)	4 (4)
*irEndocrine disorders*	5 (6)	1 (1)	–	–	–	–	5 (5)	1 (1)
Hypophysitis	4 (5)	1 (1)	–	–	–	–	4 (4)	–
Hypothyroidism	1 (1)	–	–	–	–	–	1 (1)	–
*irHepatic disorders*	4 (5)	1 (1)	–	–	–	–	4 (4)	1 (1)
Increased ALT	1 (1)	–	–	–	–	–	1 (1)	–
Increased AST	1 (1)	–	–	–	–	–	1 (1)	–
Other	2 (2)	1 (1)	–	–	–	–	2 (2)	1 (1)

*ir* immune related, *GI* gastrointestinal, *ALT* alanine aminotransferases, *AST* aspartate aminotransferases, *MUP* melanoma of unknown primary

^a^Patients may have had more than one adverse event

^b^Other gastrointestinal disorders were abdominal pain (n = 6 grade 1/2; n = 3 grade 3/4), constipation (n = 1 grade 1/2) and elevated lipase (n = 1 grade 3/4)

## Discussion

This prospective DeCOG phase II trial evaluated the efficacy and safety of ipilimumab in a cohort of 103 patients with 83 pretreated metastatic cutaneous, seven mucosal and 13 occult melanoma. The distribution rate of these clinical subgroups in our trial—considered as representative for a daily routine hospital setting—has been very similar to the rates reported for a named-patient program in Germany with approximately 200 patients [14] [Data not disclosed]. In both multi-center studies, patients with pretreated cutaneous melanoma represented approximately 80 % of all patients; patients with mucosal melanoma (DeCOG: 7 %; expanded access program (EAP) Germany: 5 %) and with occult melanoma (DeCOG: 13 %; EAP Germany: 11 %) were enrolled less frequently. Very similar distribution rates were also reported from large EAPs with 3 mg/kg ipilimumab in Italy, Spain and Australia [15–17] (Additional file 4: Table S4), with rather constant percentages of mucosal melanoma patients (ranging from 7 to 8 %) and occult melanoma patients (6–8 %) enrolled.

The reported OS rate at 12 months of 38 % for patients with cutaneous melanoma is fitting with data from several other studies (range 33–38 %) (Additional file 4: Table S4) enrolling daily clinical routine patients with a high portion of this melanoma subgroup [14–17]; a report on an EAP run in the Netherlands and the UK with cutaneous melanoma patients only also resulted into an 1-year OS rate of 38 % [18]. In a pivotal, randomized phase II dose-ranging study, in which patients with ocular and mucosal melanoma were excluded as well as patients with brain metastases, the OS rate at 12 months for the ipilimumab 3 mg/kg arm of a similar size was 39 % [19]. 2-year survival rates in our study (22 %), in the Dutch-UK expanded access cohort (23 %) [18] and in the pivotal phase II study (24 %) [19] were very similar too: these rates also match with recently published data from a pooled analysis of long-term survival data from approximately 5000 patients included in the ipilimumab pivotal clinical trials and the EAP [20]. This landmark analysis depicts a plateau in the iplilimumab survival curves at around 20 %.

Compared to skin melanoma, primary noncutaneous melanomas show a less favorable outcome when treated with 3 mg/kg ipilimumab. For occult and mucosal melanoma we found lower 1-year survival rates with 27 and 14 %, respectively. Due to the very low patient numbers, the figures for mucosal melanoma are difficult to interpret. However, similar findings were reported from the Australian EAP [17]—the only other study so far reporting OS outcomes for cutaneous versus noncutaneous melanoma treated with 3 mg/kg ipilimumab. Here, the median OS for cutaneous melanoma was twice as high (11.7 months) as for patients with uveal (5.7 months) or mucosal (5.8 months) melanoma. Again, the low number of patients with noncutaneous melanoma resulted into very huge confidence intervals. In the Italian EAP, a median OS of 6.4 months was reported for 71 patients with mucosal melanoma [21]—a value slightly below the 6.8 months reported for the overall patient collective of around 850 patients, 74 % of them with cutaneous melanoma [15]. However, survival data for cutaneous melanoma patients only were not reported from Italy. A retrospective case series from the US similarly found a median OS of 6.4 months for a cohort of 34 mucosal melanoma patients [22]. These estimates all remain below the median OS of 10–11 months, reported for primarily cutaneous melanoma patient collectives in the pivotal phase III trial with highly selected patients and a retrospective long-term-survival landmark analysis [6, 20]. The observed OS difference may be explained by the acknowledged aggressive character of mucosal melanoma; this clinical subgroup represents distinct clinicopathological and molecular features linked with reduced survival rates [10, 21]. For the 13 patients with occult melanoma, data for comparison of efficacy are not available from other studies (Additional file 4: Table S4). Due to the small sample, the inconclusive outcomes in terms of median overall survival and 1-year survival rate do not allow any conclusion, although a better survival outcome for stage IV patients with nodal metastasis of melanoma from an unknown primary (MUP) versus melanoma from a known primary has been reported in a retrospective cohort study [23]. The relative high portion of occult melanoma patients in our initial study cohort, as compared to literature [24, 25], is considered as a selection effect, because such patients usually cannot be included into clinical trials.

In our study, four doses of ipilimumab, the absence of brain metastases, and an ALC ≥1000/µl at week 4 were identified as factors independently associated with a better OS in the 83 patients with cutaneous melanoma. These findings enforce the current level of evidence gained by several studies that the completion of the four-dose-induction phase [14, 26], the absence of brain metastases [14–16], and high ALC counts and/or changes in ALC pharmacodynamics [16–18, 26–29] are predictive for a significant prolongation of survival of ipilimumab-treated patients. Investigations continue to further clarify the role of ALC as an on-treatment pharmacodynamic marker of ipilimumab activity. However, biomarkers to select upfront the right patients for ipilimumab use are still missing. The identification of an immunological biomarker during the development of the anti PD-1 inhibitor nivolumab [30, 31] and the subsequent validation of PD-L1 expression in the course of the pivotal phase III trials [2, 32–34] documents the potential and usefulness of such an approach. Ultimately, melanoma patients are expected to further benefit from a combination of such immunological treatment approaches, administered either sequentially [2, 4, 5] or concomitantly [35]. Furthermore, two PD-1 inhibitors, pembrolizumab [34] and nivolumab [33], as well as the combination of nivolumab and ipilimumab have been shown to improve the progression-free [33, 34] and overall survival [34] compared with ipilimumab in phase 3 clinical trials in patients with metastatic melanoma.

Similar to previous studies of ipilimumab at a dose of 3 mg/kg [6, 36] immune-related dermatological AEs, i.e. pruritus and rash, and immune-related gastrointestinal AEs, i.e. diarrhea and colitis were the most frequent treatment-related adverse events. The rate of grade 3 and 4 treatment-related AEs in patients with cutaneous melanoma were in line with the results of the pivotal phase III trial of ipilimumab [6]. Most of the irAEs were reversible when managed as per protocol-specific treatment guidelines and resolved with systemic glucocorticosteroid therapy. No new and unexpected safety findings were noted except one death with unknown cause was reported and the causal relationship to ipilimumab could not be excluded as per investigator.

Our phase II trial was limited by several factors; (1) the single-arm, non-randomized phase II design, however, at the time of study enrollment, no clear standard therapy for pretreated metastatic melanoma, especially for metastatic mucosal melanoma existed, (2) the small sample sizes of patients with mucosal and occult melanoma, (3) the lack of central review of imaging studies, and (4) the missing classification of tumor assessments according to immune-related response criteria [37].

## Conclusions

In conclusion, ipilimumab is a treatment option for patients with advanced cutaneous melanoma seen in daily routine. Given the small number of patients with metastatic mucosal and occult melanoma, it is not possible to determine whether ipilimumab has activity in these melanoma subgroups. The ALC at week 4 appears to be an early biomarker of response and need further confirmation in randomized controlled trials. Immune-related AEs were manageable and reversible in most of the cases.

## Additional files

10.1186/s12967-015-0716-5 Consort Diagram.
10.1186/s12967-015-0716-5 Protocol DeCOG –MM-PAL11-Trial.
10.1186/s12967-015-0716-5 Patients with different response pattern in intracranial and extracranial metastases.
10.1186/s12967-015-0716-5 Summarized data^a^ reporting the efficacy of ipilimumab 3 mg/kg in clinical-practice-settings incl. EAP.
