# Supplementary material for: Open-label, multicenter, single-arm phase II DeCOG-study of ipilimumab in pretreated patients with different subtypes of metastatic melanoma
Source: J Transl Med. 2015 Nov 6;13:351. doi: 10.1186/s12967-015-0716-5 (PMC4635983; doi:10.1186/s12967-015-0716-5)
Supplement: Supplementary file 1 — 10.1186/s12967-015-0716-5 Consort Diagram. [file 12967_2015_716_MOESM1_ESM.docx]

**Additional Figure S1: Consort Diagram**

**Part 1** (May 20, 2011 to August 31, 2011):

n=108 patients with cutaneous, mucosal and occult metastatic melanoma

n=13 patients with metastatic ocular melanoma (reported elsewhere)

Part 2 (October 1, 2011 to September 30, 2012):

n=42 patients with metastatic ocular melanoma (as reported elsewhere)

Completed the induction phase (4 doses of ipilimumab) (n=64)

Off Treatment (n=39)

- Disease Progression (n=16)
- Death (n=11)
- Toxicity (n=8)
- Withdrew informed consent (n=4)

Evaluable for tumor assessment (n=70)

Part 1 not treated (n=5)

- Cutaneous (n=3)
- Occult (n=2)

**Part 1**:

n=103 patients with metastatic cutaneous (n=83), mucosal (n=7) and occult (n=13) melanoma received at least one dose of ipilimumab 3mg/kg

Not evaluable for tumor assessment (n=33)

- Death (n=22)
- Disease Progression (n=3)
- No measurable disease at baseline (n=1)
- Toxicity (n=3)
- Withdrew informed consent (n=3)
- Lost to follow up (n=1)

Lost to follow up (n=1)

Follow up (n=102)
