# Supplementary material for: Open-label, multicenter, single-arm phase II DeCOG-study of ipilimumab in pretreated patients with different subtypes of metastatic melanoma
Source: J Transl Med. 2015 Nov 6;13:351. doi: 10.1186/s12967-015-0716-5 (PMC4635983; doi:10.1186/s12967-015-0716-5)
Supplement: Supplementary file 3 — 10.1186/s12967-015-0716-5 Patients with different response pattern in intracranial and extracranial metastases. [file 12967_2015_716_MOESM3_ESM.docx]

**Additional File S3:** Patients with different response pattern in intracranial and extracranial metastases

| Patient | Intracranial response | Extracranial response | Overall response |
| --- | --- | --- | --- |
| 1 | CR | PD | PD |
| 2 | SD | PD | PD |
| 3 | SD | PR | PR |
| 4 | SD | PD | PD |
| 5 | SD | PR | PR |

Abbreviations: CR, complete response; PR, partial response; SD, stable disease; PD, progressive disease.
