# Supplementary material for: Open-label, multicenter, single-arm phase II DeCOG-study of ipilimumab in pretreated patients with different subtypes of metastatic melanoma
Source: J Transl Med. 2015 Nov 6;13:351. doi: 10.1186/s12967-015-0716-5 (PMC4635983; doi:10.1186/s12967-015-0716-5)
Supplement: Supplementary file 4 — 10.1186/s12967-015-0716-5 Summarized dataa reporting the efficacy of ipilimumab 3 mg/kg in clinical-practice-settings incl. EAP. [file 12967_2015_716_MOESM4_ESM.docx]

**Additional Table S4: Summarized data^a^ reporting the efficacy of ipilimumab 3 mg/kg in clinical-practice-settings incl. EAP**

|  | **Study type** | **Treatment** | **Patient characteristics (Stage, N prev. ther., ECOG status, AE**/**SAE)** | **Melanoma Subtype** | **Patients N (*%*)** | **Med. OS (mts) (95% CI)** | **1-yr OS rate (95% CI)** | **2-yr OS rate (95% CI)** | **Med. PFS (mts) (95% CI)** | **ORR**  **(*%*)** |
| --- | --- | --- | --- | --- | --- | --- | --- | --- | --- | --- |
| Zimmer L et al, *PLoS One* 2015, **10**:e0118564 | | | |  |  |  |  |  |  |  |
|  | Phase II | Ipi 3 mg q3w | All Stage IV (M1a-M1c), CTx-naïve & pre-treated pts, ECOG 0-2^b^ **🡪 AE/SAE: 35 pts (*66%*) / 19 (*36%*)** | Uveal | **53** | 6.8 (3.7-8.1) | *22* (*12-31*) | *7* (*1-18*) | 2.8 (2.5-2.9) | *0* |
| Eigentler T et al, *J Immunother* 2014, **37**:374-381 | | | |  |  |  |  |  |  |  |
|  | EAP (DE) | Ipi 3 mg q3w | All Stage IV (M1a-M1c), CTx-naïve (4%) & pre-treated pts, ECOG 0-2  **🡪 AE/SAE: 67 pts (*34%*) / 24 (*12%*)** | **All** | **198** | 6.8 (5.6-10.3) | *38* |  |  | *11* |
|  |  |  |  | Uveal | 13 (*7*) |  |  |  |  |  |
|  |  |  |  | Mucosal | 10 (*5*) |  |  |  |  |  |
|  |  |  |  | Occult | 22 (*11*) |  |  |  |  |  |
| Ascierto PA et al. *J Transl Med* 2014, **12**:116 | | | |  |  |  |  |  |  |  |
|  | EAP (IT) | Ipi 3 mg q3w | Stage III/IV (M1a-M1c), pre-treated pts, ECOG 0-2  **🡪 AE/SAE: 399 pts (*47%*) / 100 (*12%*)** | **All** | **855** | 7.2 (6.4-8.0) | *35* | *20* | 3.7 (3.4-4.0) | *13* |
|  |  |  |  | Uveal | 83 (*10*) |  |  |  |  |  |
|  |  |  |  | Mucosal | 71 (*8*) |  |  |  |  |  |
|  |  |  |  | Occult | 70 (*8*) |  |  |  |  |  |
| Maio M et al. *Ann Oncol* 2013, **24**: 2911–2915 | | | |  |  |  |  |  |  |  |
|  | EAP (IT) | Ipi 3 mg q3w | Stage IV, pre-treated pts, ECOG 0-2  **🡪 AE/SAE: 47 pts (*57%*) / 8 (*10%*)** | Uveal | **83** | 6.0 (4.3-7.7) | *31* |  | 3.6 (2.8-4.4) | *5* |
| Del Vecchio M et al. *Eur J Cancer* 2014, **50:**121-127 | | | |  |  |  |  |  |  |  |
|  | EAP (IT) | Ipi 3 mg q3w | Stage III/IV (M1a-M1c), pre-treated pts, ECOG 0-2  **🡪 AE/SAE: 33 pts (*48%*) / 11 (*16%*)** | Mucosal | **71** | 6.4 (4.1-8.7) | *35* |  | 4.3 (3.4-5.2) | *12* |
| Berrocal A et al. *Melanoma Res* 2014, **24**:577-583 | | | |  |  |  |  |  |  |  |
|  | EAP (ES) | Ipi 3 mg q3w | All Stage IV (M1a-M1c),pre-treated pts, ECOG 0-2  **🡪 SAE: 9 (*6%*)** | **All** | **355** | 6.5 (4.1-8.9) | *33* |  |  | *11* |
|  |  |  |  | Uveal | 40 (*11*) |  |  |  |  |  |
|  |  |  |  | Mucosal | 24 (*7*) |  |  |  |  |  |
|  |  |  |  | Occult |  |  |  |  |  |  |
| Alexander M et al. *Med J Aust* 2014, **201**:49-53 | | | |  |  |  |  |  |  |  |
|  | EAP (AU) | Ipi 3 mg q3w | Stage III/IV (M1a-M1c), pre-treated pts, ECOG 0-2  **🡪 AE/SAE: 18 pts (*17%*) / 11 (*16%*)** | **All** | **104** | 9.6 (6.6-12.4) | 42 (32-52) | 18 (9-30) | 3.0 (2.7-3.4) |  |
|  |  |  |  | Cutaneous | 79 (*76*) | 11.7 (7.1-13.8) |  |  | 3.0 (2.7-3.4) |  |
|  |  |  |  | Uveal | 11 (*11*) | 5.7 (1.5-16.0) |  |  | 3.5 (0.9-7.3) |  |
|  |  |  |  | Mucosal | 8 (*8*) | 5.8 (1.1- ) |  |  | 2.7 (0.5-3.9) |  |
|  |  |  |  | Occult/unk | 6 (*5*) |  |  |  |  |  |
| Kelderman S et al. *Cancer Immunol Immunother* 2014, **63**:449-458 | | | | |  |  |  |  |  |  |
|  | EAP (NL) | Ipi 3 mg q3w | Stage III/IV (M1a-M1c), pre-treated pts, WHO 0-2  **🡪 SAE: 28 (*16%*)** | Cutaneous | **166** | 7.5 (6.1-10.5) | *38 (31-46)* | *23 (16-32)* | 2.9 (2.8-3.2) | *17* |
| Kelderman S et al. *Acta Oncol* 2013 **66**:1786-1789 | | | |  |  |  |  |  |  |  |
|  | Phase II | Ipi 3 mg q3w | Stage III/IV (M1a-M1c), pre-treated, WHO 0-2  **🡪 SAE: 3 (*14%*)** | Uveal | **22** | 5.2 (4.9-5.6) | *27* |  | 2.9 (2.3-5.3) | *9* |
| Postow MA et al. *Oncologist* 2013, **18**:726-732 | | | |  |  |  |  |  |  |  |
|  | EAP (US) + hosp. charts | Ipi 3 mg q3w (26 pts only^c^) | Stage III/IV, CTx-naïve (24%) & pre-treated pts, ECOG 0-2 | Mucosal | **33** | 6.4 (1.8-26.7) |  |  |  | *7* |
| Luke JJ et al. *Cancer* 2013, **119**: 3687–369 | | | |  |  |  |  |  |  |  |
|  | EAP (US) monocentric | Ipi 3 mg q3w (34 pts only^c^) | All Stage IV (M1a-M1c), CTx-naïve & pre-treated pts, ECOG 0-2  **🡪 AE/SAE: 23 pts (*68%*) / 5 (*15%*)** | Uveal | **39** | 9.6 (6.3-13.4) |  |  |  | *5* |

Abbreviations: AE, adverse events; CTx, chemotherapy; EA, Early Access Program; ECOG, Eastern Cooperative Oncology Group; Ipi, ipilimumab; mts, months; PFS, progression-free survival; ORR, overall response rate; OS, overall survival; SAE, serious adverse events; unk, unknown

^a^ Data as reported in the respective publication. Free text fields indicate that either no data were reported or data were reported in a different format

^b^ Only 1 patient with ECOG 2 status

^c^ The other patients received ipilimumab 10 mg/kg q3w
